# Antiviral properties of porous graphene, graphene oxide and graphene foam ultrafine fibers against Phi6 bacteriophage

**DOI:** 10.3389/fmed.2022.1032899

**Published:** 2022-11-24

**Authors:** Seda Gungordu Er, Tanveer A. Tabish, Mohan Edirisinghe, Rupy Kaur Matharu

**Affiliations:** ^1^Department of Mechanical Engineering, University College London, London, United Kingdom; ^2^Radcliffe Department of Medicine, University of Oxford, Oxford, United Kingdom; ^3^Department of Engineering Science, University of Oxford Begbroke Science Park, Oxford, United Kingdom; ^4^Department of Civil, Environmental and Geomatic Engineering, University College London, London, United Kingdom

**Keywords:** graphene oxide, porous graphene, graphene foam, antiviral, nanofiber, electrospinning, Phi6 bacteriophage

## Abstract

As the world has experienced in the Coronavirus Disease 2019 pandemic, viral infections have devastating effects on public health. Personal protective equipment with high antiviral features has become popular among healthcare staff, researchers, immunocompromised people and more to minimize this effect. Graphene and its derivatives have been included in many antimicrobial studies due to their exceptional physicochemical properties. However, scientific studies on antiviral graphene are much more limited than antibacterial and antifungal studies. The aim of this study was to produce nanocomposite fibers with high antiviral properties that can be used for personal protective equipment and biomedical devices. In this work, 10 wt% polycaprolactone-based fibers were prepared with different concentrations (0.1, 0.5, 1, 2, 4 w/w%) of porous graphene, graphene oxide and graphene foam in acetone by using electrospinning. SEM, FTIR and XRD characterizations were applied to understand the structure of fibers and the presence of materials. According to SEM results, the mean diameters of the porous graphene, graphene oxide and graphene foam nanofibers formed were around 390, 470, and 520 nm, respectively. FTIR and XRD characterization results for 2 w/w% concentration nanofibers demonstrated the presence of graphene oxide, porous graphene and graphene foam nanomaterials in the fiber. The antiviral properties of the formed fibers were tested against *Pseudomonas phage* Phi6. According to the results, concentration-dependent antiviral activity was observed, and the strongest viral inhibition graphene oxide-loaded nanofibers were 33.08 ± 1.21% at the end of 24 h.

## Introduction

Viruses are nanosized obligate intracellular parasites that need a living host cell to survive and reproduce (1). They cause viral infections which can result in a significant level of morbidity and mortality, like the current Coronavirus Disease 2019 (COVID-19) pandemic threatening the world (2, 3). There are a number of ways to treat viral infections, such as developing vaccines and antiviral drugs, however, some viruses can overcome these treatments because they mutate rapidly (4). Thus, people are primarily encouraged to prevent contamination by using personal protective equipment (PPE) (5–7). Transmissibility of a virus depends on the virus variation, and the routes of transmission might be divided into direct (person-to-person) contact, indirect (object-to-individual) contact, droplets and aerosols (8). Adenovirus, enterovirus, metapneumovirus, rhinovirus (RV), influenza, respiratory syncytial virus (RSV) and coronavirus are among the pathogens that cause respiratory tract infections (9). Severe acute respiratory syndrome coronavirus 2 (SARS-CoV-2), the cause of COVID-19 emerged in China and spread rapidly around the world, consequently affecting millions of people (3). In this challenging time, face masks, hand sanitizing and social distancing have been recommended by the World Health Organization (WHO) and governments as the first step in a comprehensive prevention strategy to suppress COVID-19 transmission and save lives (10). It has been reported by researchers that SARS-CoV-2 can maintain its viability in aerosols for up to 3 h (11, 12). Microdroplets emitted into the air when coughing, talking, or breathing are the main sources of airborne transmission of these viruses (13). Bacteriophage Phi6 belongs to the *Cystoviridae* family and infects *Pseudomonas* bacteria. Similar to SARS-CoV-2 it is enveloped by a lipid membrane, has spike proteins, and is of similar size (80–100 nm), thus making it a good surrogate for studying RNA viruses (14, 15).

Graphene is a two-dimensional (2D) honeycomb shape lattice of carbon atoms that was initially prepared by micromechanical cleavage of bulk graphite (16, 17). Graphene has emerged as one of the most promising nanomaterials that have attracted the scientific community’s interest because of its unique combination of extraordinary properties such as high electrical and thermal conductivity, high purity, good bio-functionalizability, solubility, the capability of easy cell membrane penetration for high antimicrobial effects, high surface area and theoretical strength (18–22). These unusual properties of graphene offer a fascinating material platform in biomedical research like wearable electronics (23), ultrasensitive biosensors (24, 25), tissue engineering (26, 27) and antimicrobial filtration (18–20). Polymer composite fibers containing antimicrobial agents (graphene derivatives, copper (Cu), curcumin, chitosan, tellurium, titanium dioxide (TiO_2_), etc.) play a crucial role in the development of PPE, wound dressing films and filters to reduce the level of microbial contamination and bioburden (28–32).

The antimicrobial activity of graphene and its derivatives is due to a combination of physicochemical properties. These can be listed as oxidative-stress mediated, layer number, lateral size effect, tailored surface, time and concentration dependency (33–36). Oxidative stress mediated antimicrobial activity containing functional groups (hydroxyl, epoxy, carboxyl) is generally seen in graphene oxide (GO) and reduced graphene oxide (rGO), as functional groups increase, the antimicrobial effect also increases (37). Since graphene has sharp edges, it is expected that the antimicrobial activity will decrease as the lateral size increases (38). The antimicrobial effect will also increase as the layer number increases (38). Additionally, agglomeration of graphene nanomaterials gives rise to antimicrobial activity, as it reduces microbial interaction and prevents nutrients from reaching the microbes (39). The increase in the exposure time and the dose of the material also increases the antimicrobial activity.

Many studies have been reported on graphene and its derivatives and their nanocomposites discussing antibacterial, antifungal and antiviral activities (18–20, 40–42). Matharu et al. investigated the antibacterial activity of 2, 4 and 8 w/w% concentration graphene nanoplatelet (GNP) and GO loaded polymethylmethacrylate (PMMA) composite nanofibers against *Escherichia coli (E. coli)* (19, 20). According to these dose-dependent results, GO and GNP loaded with 8 w/w% showed the highest antimicrobial efficiency, around 85% and 95% respectively. Likewise, in another study, the antibacterial properties of GO added to polyurethane (PU) polymer was observed at different concentrations (0, 1, 5, 10 wt%) (43). It was concluded that *E. coli* and *Staphylococcus aureus (S. aureus)* bacteria decreased the at most at the highest concentration level (10 wt%). Since this study was conducted for water purification application, it is not a concern of toxic effects on the human body, but it is essential for graphene-based materials for biomedical research.

**FIGURE 1:**
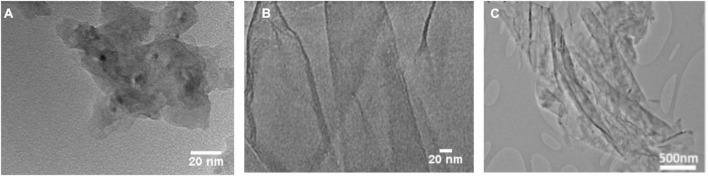
High-resolution transmission electron microscopy characterization of **(A)** PG **(B)** GO **(C)** GF. In **(C)** has been reproduced with permission from reference (48) Copyright 2017, Materials.

GO, porous graphene (PG) and graphene-based foam (GF) are among the graphene derivatives (Figure 1) sought after. PG refers to graphene-related materials that have nano-sized pores on the basal plane, the size and distribution of which differ according to the synthesis method. PG, which has structural properties very close to pristine graphene, can be synthesized by chemical and physical means (44). It is used especially for gas separation and purification applications paying attention to its high permeability (44, 45). PG polymer composite nanofibers have been formed using pressurized gyration and the surface topography was studied by Ahmed et al. (40). According to this study, PG was seen as a promising material in ultrafiltration applications (40). GO is a material that many researchers have examined, especially its antibacterial activity as mentioned above (20, 46, 47). GO contains functional groups causing high levels of oxidative stress that play a major role in its antimicrobial effect against pathogens (33). Matharu et al. reported the antiviral activity of GO, here the mechanism could be a physical and chemical interaction and GO virucidal action increases depending on time and concentration (18). GF is a graphene derivative with a high surface area providing a uniform and homogeneous distribution of graphene in biomedical applications (48, 49). Unlike PG and GO, GF has a 3-dimensional structure and it has low density. Wang et al. reported that GF shows significant biomineralization in engineering, scaffolds formed from the GF-polycaprolactone (PCL) composite are a good example (49). No significant cytotoxicity was found in liver and kidney macrophages for 7 days, according to GF biocompatibility and toxicity assessments (48).

In this study, the antiviral activity of electrospun PG, GO and GF nanocomposite fibers were assessed against a double-stranded RNA virus. Electrospinning is one of the most common fiber production methods used to obtain nano-sized polymeric fibers from various polymer solutions applying high voltage. PCL was used as the carrier polymer as it is a biocompatible, easily processable polymer, and when it is dissolved in acetone, a non-toxic environmentally friendly solution is obtained when used in the human body. Previous antimicrobial studies of graphene were mostly focused on the antibacterial and viral inhibition was not included in much research. At the same time, the antiviral activity of PG and GF are investigated for the first time in this study. These comparative antiviral fibrous structures enable us to find the optimum material for PPE for future antiviral filtration systems.

## Materials and methods

### Materials and preparation of solutions

PCL (Mw ∼ 80,000 g/mol), GO (average number of layers 15–20) and acetone were purchased from Sigma Aldrich (Gillingham, UK). PG (pore size around 3–5 nm) and GF (sized about 4 μm with folded area and number of layers varied from 2 – 3 to 9 – 15) were synthesized as reported by Tabish et al. in the previous research (40, 48, 50).

**FIGURE 2:**
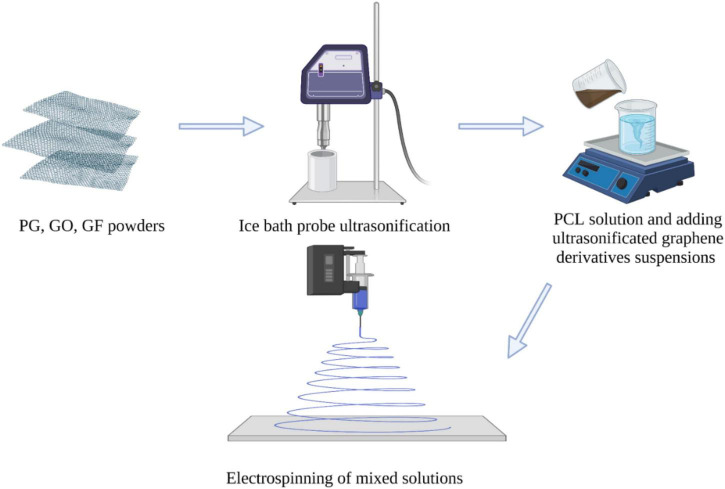
Schematic demonstrating the production of PG, GO and GF-based polymer composite fibers with electrospinning. All images within this figure are prepared in Biorender.com and have been used with permission from Biorender.

PG, GO, GF powders and PCL polymer (10 wt%) were weighted on precision scales for 5 different concentrations (0.1, 0.5, 1, 2, 4 w/w%) determined as indicated in Table 1, and were suspended into acetone solvent. The solutions were prepared in two separate parts and mixed at the end. The first part is PCL and acetone solution, and the second part is nanomaterial and acetone suspension. PG, GO and GF nanomaterials were calculated as in the table and added to the acetone solvent. The PG, GO and GF suspensions were then sonicated in an ice bath to achieve a homogenized solution with a Branson SFX 550 Digital Probe Sonifier (Cole-Parmer, Eaton Socon, UK) set at 80–100% for approximately 2 h and then left overnight on a magnetic stirrer (Figure 2). In order for the PCL pellets to dissolve in acetone, they were left overnight on a heated magnetic stirrer set at 50°C. After the polymers were completely dissolved, they were mixed with the homogenized suspension solutions after ultrasonication was completed. Then all solutions were left on the magnetic stirrer for ∼4 h.

**TABLE 1:** Composition of porous graphene, graphene oxide and graphene foam loaded polycaprolactone (PCL) nanofiber solutions.

Final concentration (w/w%)	PCL (g)	Solvent (acetone) (mL)	PG (g)	Solvent (acetone) (mL)	GO (g)	Solvent (acetone) (mL)	GF (g)	Solvent (acetone) (mL)
0	5	50	–	–	–	–	–	–
0.1	5	25	0.005	25	0.005	25	0.005	25
0.5	5	25	0.025	25	0.025	25	0.025	25
1	5	25	0.05	25	0.05	25	0.05	25
2	5	25	0.1	25	0.1	25	0.1	25
4	5	25	0.2	25	0.2	25	0.2	25

### Surface tension and viscosity

Surface tension measurement of 15 different nanocomposite solutions was performed using Kruss Tensiometer (Tensiometer K9, Hamburg, Germany). During this measurement using the Du Nouy ring method, approximately 10 mL of solution was taken from the glass bottle and a platinum-iridium ring was dipped into it and slowly withdrawn. In order to ensure correct results, the ring was first calibrated with distilled water and then used for solutions. The maximum surface tensions obtained during extraction were recorded. This process was performed three times for each solution and the averages were recorded. The ambient temperature was recorded as approximately 23°C.

A Brookfield Viscometer DV-III was used to determine the viscosity of the solutions (Brookfield, Middleboro, MA, USA). For each of the solutions, approximately 6 ml was poured into the viscometer and the values measured with a small-sample spinner were recorded. Viscosity measurements of nanocomposite solutions were carried out at ambient conditions (23°C) and were performed three times to get an average result.

### Fabrication and characterization of loaded fibers

PG, GO, and GF incorporated fibers were collected using a grounded metal flat plate collector (Figure 2). The needle used was 18G (1.25 mm BD micro lance) and the capillary tube was polytetrafluoroethylene (PTFE) (outer diameter 2 mm and inner diameter 1 mm). DC power supply provides an applied voltage of 12–16 kV. The distance between the needle tip and the collector was 150 mm, and the flow rate was set at 0.2 mL/min. The humidity in the room during electrospinning was recorded in the range of 45–53%, the temperature was measured in the range of 23–26°C.

The fibers were gathered after manufacture, mounted on aluminum studs, and gold sputter coated for 90 s (Q150R ES Quorum Technologies Ltd., Laughton, UK). After that, these samples were examined using a scanning electron microscope (SEM) (Hitachi S-3400n, Tokyo, Japan) with a 5 kV working voltage. The morphology of the nanocomposite fiber mats were determined using SEM at about 10 different areas of a sample. A total of 100 fibers were randomly measured using Image J software, and the mean diameter and standard deviation were computed using Excel. OriginPro software was used to create the histogram graphs of the frequency distribution of fiber diameters.

Fourier transform infrared spectroscopy (FTIR) (Bruker Optics Tensor-27 IR, Ettlingen, Germany) characterization with a wavelength range of 4,000–500 cm^–1^ was applied to pure PCL and 2w/w% PG-PCL, GO-PCL, GF-PCL nanofibers. Nanofiber samples were adjusted to be approximately 2 mm thick and 5 mm in diameter and placed in the spectrometer. X-ray diffraction (XRD) (at 40 kV and –40 mA) characterization was used for 4 different samples at the same concentration.

### Antiviral studies

In this work, *Pseudomonas* Phi6 bacteriophage was used to model double-stranded RNA viruses. *Pseudomonas syringae* (*P. syringae)* and *Pseudomonas* Phi6 bacteriophage were sourced from DSMZ (Braunschweig, Germany). The received microorganisms were cultured following the manufacturer’s instructions. Stock cultures of *P. syringae* were stored in a Microbank™ at −20°C, whilst the Phi6 bacteriophage was stored in a cool dark place. Antiviral activity was assessed against this microorganism as it is a safe, easy to work with and well-studied model surrogate for SARS-CoV-2.

Actively growing broth cultures of *P. syringae* were prepared by incubating a single colony in 30 ml of tryptone soya broth for 24 h at 25°C and 150 rpm.

Bacteriophage suspensions containing the made fibers in PBS were prepared. A total of 100 μl of the suspension at 0 and 24 h was added to 300 μl of the overnight *P. syringae* culture and 3 ml of molten semi-solid agar (0.5% agar) and poured onto agar plates. The plates were incubated for 24 h at 37°C and the number of plaques was counted. The viral reduction was calculated by comparing the number of virions at 24–0 h. Antiviral activity was statistically analyzed and compared to the control samples using unpaired *t*-tests. The difference was considered significant when *p* < 0.05.

## Results and discussion

### Suspension behavior

Graphene suspensions were prepared in different concentrations (0.1, 0.5, 1, 2, 4 w/w%) as PCL-PG, PCL-GO and PCL-GF. The fibers obtained from these suspensions were formed by the electrospinning method, which is affected by the surface tension and viscosity values of the solution.

**FIGURE 3:**
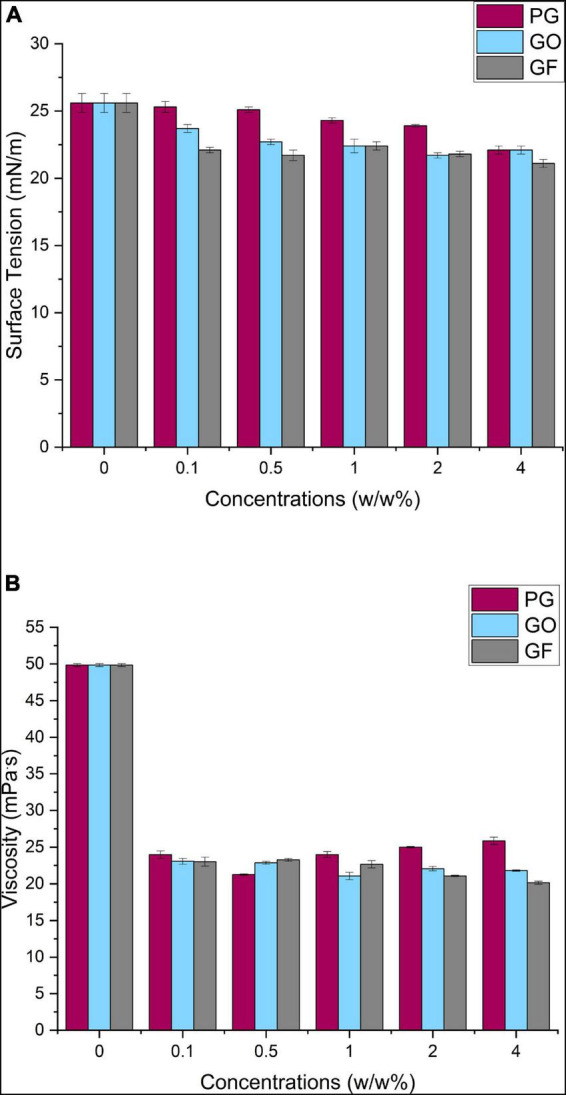
PCL-PG, PCL-GO and PCL-GF solutions surface tension **(A)** and viscosity **(B)**.

In Figure 3A, the surface tension values of the pure PCL solution and nanocomposite solutions loaded with PG, GO and GF at increasing concentrations are shown. According to the results, the pure PCL solution has relatively higher surface tension than the fluids with PG-GO-GF added. The range of decrease was observed as approximately ∼1.3% for PCL-PG, ∼3.9% for PCL-GO and ∼3.4% for PCL-GF at different concentrations. The pure PCL solution had an average surface tension of 25.6 ± 0.7 mN/m, this decreased to around 25.3 ± 0.4 mN/m upon the addition of 0.1 w/w% PG, however, this value did not change much with the increase of the concentration and showed a stable behavior. Likewise, the surface tension of 0.1 w/w% GO and GF dropped to 23.7 ± 0.3 and 22.1 ± 0.4 mN/m respectively, and then near-constant values were observed with increasing concentrations. According to the results in this study and literature, the addition of surfactants result in a reduction in surface tension (51). Similarly, graphene-based materials act as a surfactant and the electric force between the particles causes surface tension reduction in this study. Surface tension has a counteracting effect, and a lower voltage is required for jet initiation when surface tension is reduced. Surface tension also has a direct impact on the generation of beads (51).

The viscosity effect of graphene materials added to the PCL solution is shown in Figure 3B. Initially, the viscosity of the pure PCL solution was approximately 49.84 ± 0.2 mPa.S and a moderate decrease was observed with the addition of graphene derivatives. While the viscosity values of the suspensions with PG added were 23.99 mPa.s on average, this value slightly decreases to 23.07 and 23.2 mPa.S for GO and GF, respectively. The approximate range results was 4.58 mPa.S of PCL-PG solution, 1.92 mPa.S for PCL-GO, and 3.1 mPa.S for PCL-GF at different concentrations. The decrease in viscosity may be caused by the instability of the added nanomaterial in the solution, due to the heat of the applied solvent and polymer.

### Fiber characterization

0.1, 0.5, 1, 2 and 4 w/w% PCL-PG, PCL-GO and PCL-GF nanofibers were prepared using electrospinning. The prepared fibers were imaged using SEM to understand fiber morphology. A histogram was created with fiber diameter distribution by measuring the diameter of 100 sample fibers. The formation of some large beads was observed due to the rapid evaporation of the solvent acetone used during the electrospinning process. Due to the nature of the electrospinning method, the fibers dispersed with the aid of high voltage are not uniformly aligned. However, it is observed that adding graphene nanoparticles, which are affected by high voltage with increasing concentrations, increases the entanglement of fibers. While the applied high voltage is necessary to overcome the surface tension, the electrical conductivity of the nanomaterial used is affected by this high voltage, causing asymmetric fiber formation (52).

**FIGURE 4:**
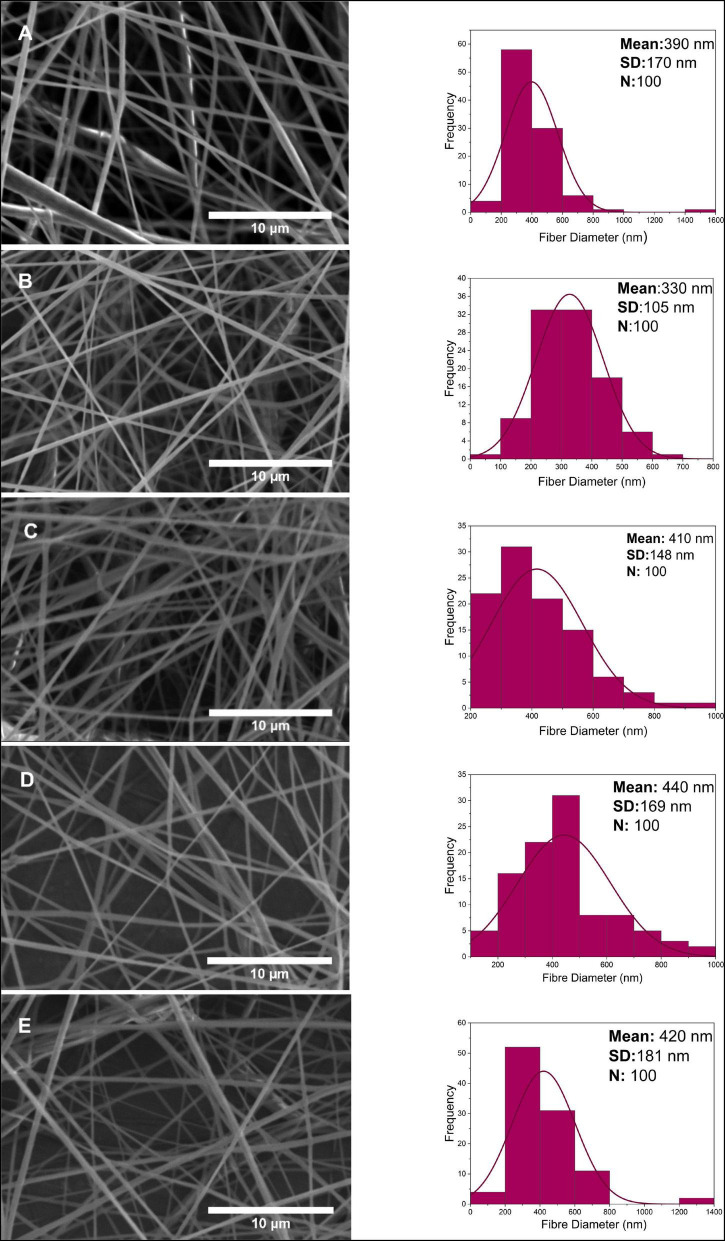
Scanning electron microscope images and fiber diameter distribution of nanofibers at 0.1, 0.5, 1, 2, 4 w/w% concentration for PG **(A–E)**.

As seen in Figure 4, PCL-PG nanofibers were tubular with some beaded, porous surface properties. The mean fiber diameter of 0.1 w/w% PG-loaded PCL was found to be approximately 390 ± 170 nm. At the highest concentration 4 w/w%, a slight diameter increase of 420 ± 181 nm was observed. Although the uniform distribution of fibers was obtained in PCL-PG polymer nanocomposite fibers in general, the thickest fiber is calculated at around 1.6 μm at 0.1 w/w% PCL-PG SEM images. While the fiber diameter for 0.1 w/w% PCL-PG was between 200 and 400 nm, it was observed that the uniformity of the other samples reduced.

**FIGURE 5:**
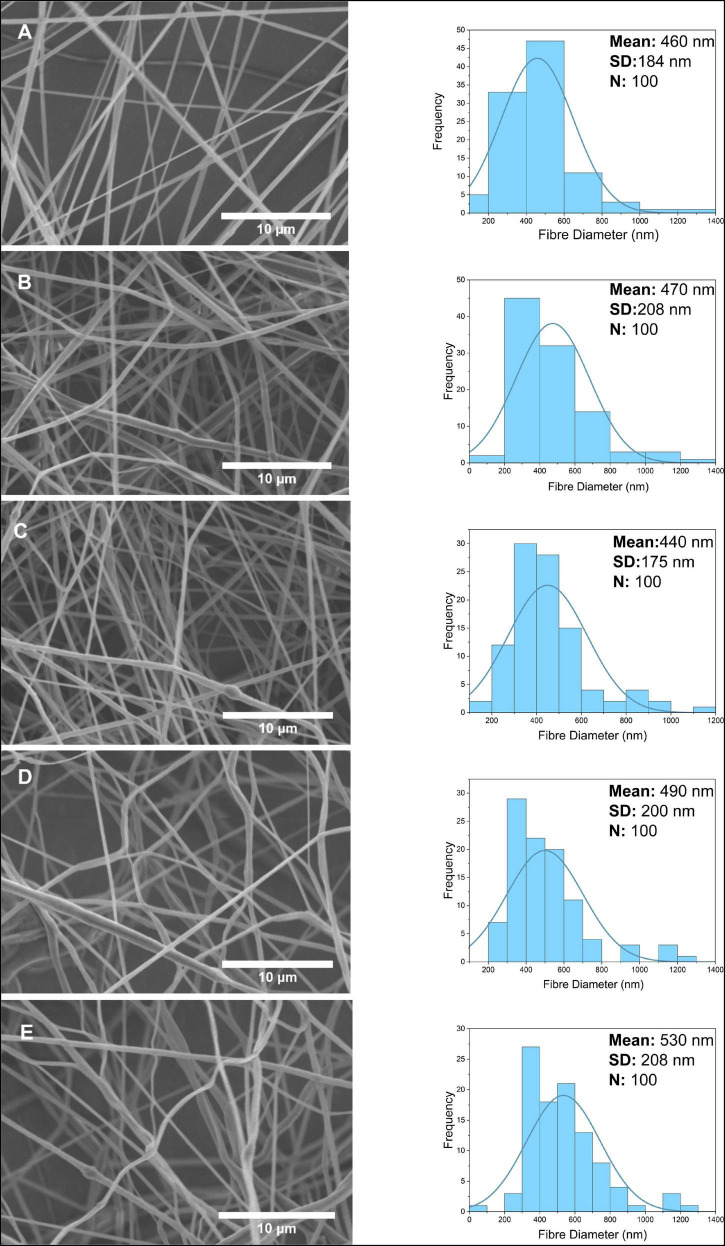
Scanning electron microscope images and *fiber* diameter distribution of nanofibers at 0.1, 0.5, 1, 2, 4 w/w% concentration for GO **(A–E)**.

SEM characterization of PCL-GO nanofibers and fiber diameter distribution is shown in Figure 5. Especially at high concentrations of PCL-GO fibrous, some bead formation is observed in SEM images. Bead formation might be related to the agglomeration of GO nanoparticles. While the dispersion of nanosized fibers showed linear alignment for 0.1 w/w% PCL-GO, GO nanoparticles affected by high voltage with increasing concentration aids curl-up of the fibers. Between the lowest and highest concentrations of GO nanofibers, the average fiber diameter was not very different, with an average of 460 ± 184 nm at 0.1 w/w% concentration, 530 ± 208 nm at 4 w/w% concentration, and no linear increase was found with increasing concentration. In general, a few thicker fibers appear above 1 μm in each GO sample diameter calculation.

**FIGURE 6:**
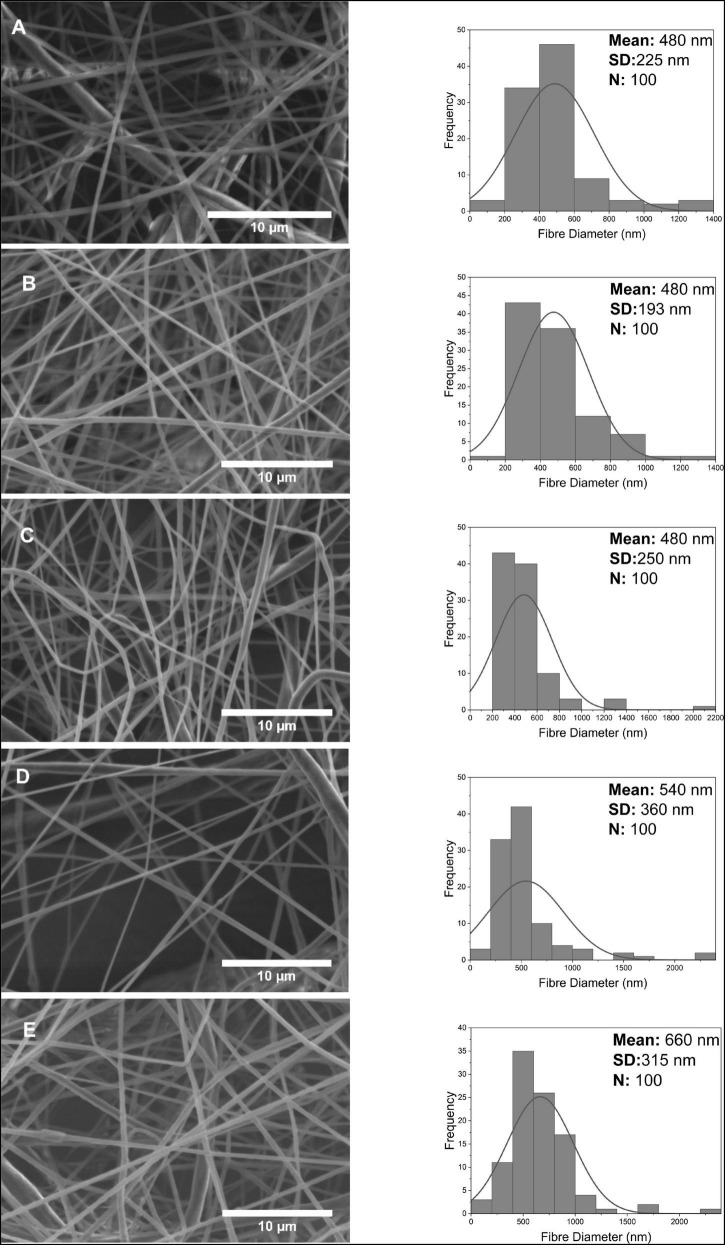
Scanning electron microscope images and *fiber* diameter distribution of nanofibers at 0.1, 0.5, 1, 2, 4 w/w% concentration for GF **(A–E)**.

The PCL-GF nanofibers shown in Figure 6 have a more porous and non-uniform structure than PG and GO. While the nanofiber diameter distribution of 0.1 w/w%, 0.5 w/w%, and 1 w/w% was almost the same, it is around 480 nm, and the mean fiber diameter moderately increased with an increasing concentration to approximately 660 ± 315 nm. Another difference in PCL-GF fibers was the standard deviations increasing with concentration and therefore decreasing uniformity. Fibers with a thickness greater than 2 μm were seen in fibers containing high concentrations of GF. The average diameters of the PG, GO and GF fibers were approximately 390, 470, and 520 nm, respectively. In summary, a mostly increase in the diameters of PG, GO and GF added nanofibers was observed depending on the concentration. This result supports that the added nanofiller increases its diameter by embedding in the fiber and/or adhering to the surface.

**FIGURE 7:**
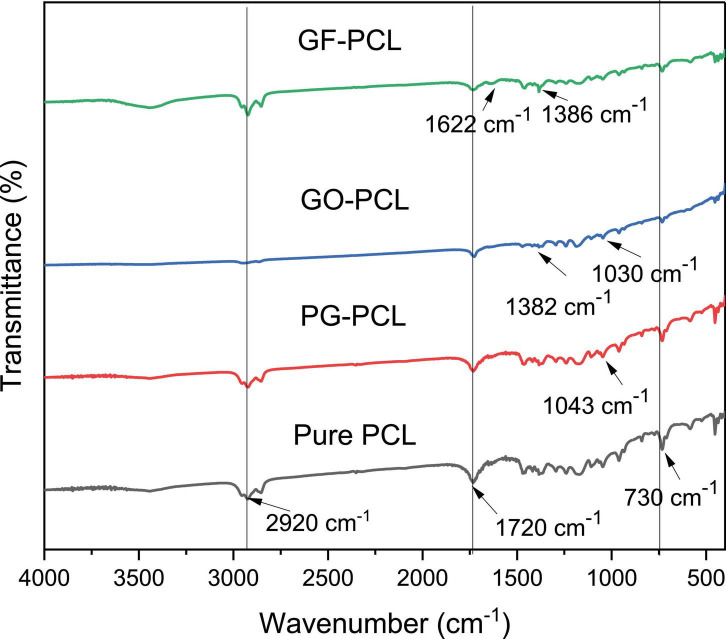
Fourier transform infrared spectroscopy analysis of pure PCL and 2w/w% PG-PCL, GO-PCL, GF-PCL nanofibers.

2 w/w% PG, GO, and GF nanofibers were analyzed by FTIR and XRD characterizations. FTIR analysis is performed to validate the presence of graphene derivatives in nanofibers. FTIR analysis of PG (40, 45), GO (45, 50, 53) and GF (48, 54) nanomaterials were performed in previous studies. FTIR peaks of pure PCL nanofiber were noticed at 2920, 1720, and 730 cm^–1^ (Figure 7). These peaks also existed in the case of other PCL-based nanofibers. The 1043 and 1720 cm^–1^ peaks encountered in the PG-PCL nanofiber correspond to the epoxy and carbon functional groups. These peaks and their corresponding functional groups are in consistent with previously published work (40). Similarly, GO-PCL nanofiber FTIR analysis generated peaks of carboxyl groups at 1,382 and 1030 cm^–1^, as shown in previous studies (45, 53). Finally, the peaks at 1622 and 1386 cm^–1^ appeared in the case of GF-PCL nanofiber typically correspond to the presence of GF (48, 54). In summary, FTIR analysis results proved the existence of PG, GO and GF nanomaterials on sample nanofibers.

**FIGURE 8:**
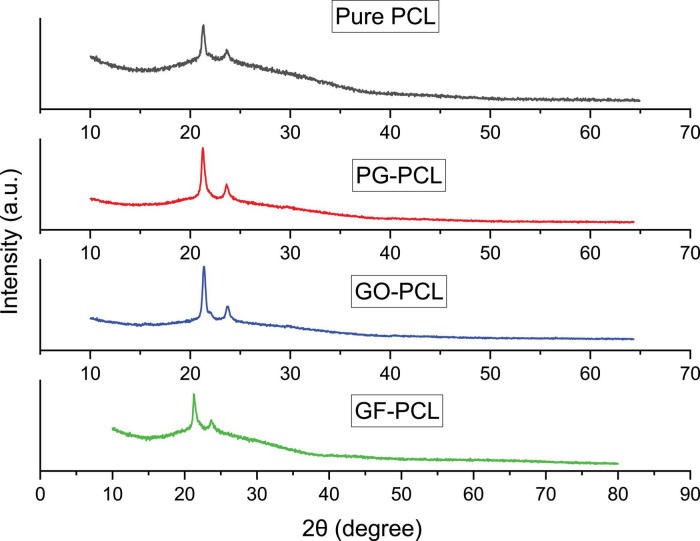
XRD analysis of pure PCL and 2w/w% PG-PCL, GO-PCL, GF-PCL nanofibers.

XRD results of pure PCL and graphene-PCL nanofibers are shown in Figure 8. Two distinct peaks are clearly seen in each graph. Since the XRD analysis results of pure PCL and other nanomaterials were similar, no significant shift in peaks was observed indicating the presence of PG, GO, and GF. These results can be interpreted as the fiber crystallization process is similar.

### Antiviral activities

The viricidal properties of the nanocomposite fibers were tested against bacteriophage Phi6. A plaque assay was used to quantify the number of infectious viral particles in suspension before and after treatment. The advantage of using plaque assays is their ability to give a direct quantitative measurement of the exact number of virions in suspension (55, 56).

**FIGURE 9:**
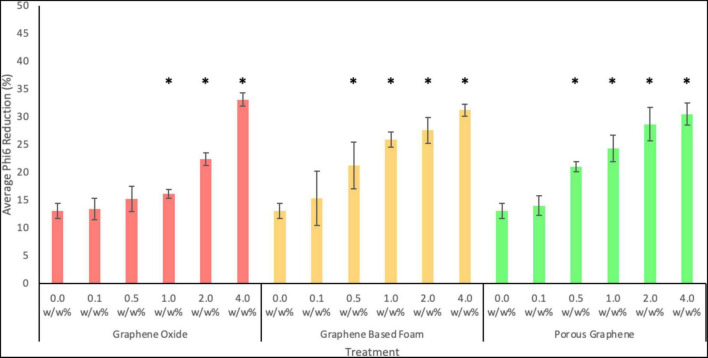
Antiviral activity of PCL fibers loaded with 0.1, 0.5, 1.0, 2.0 or 4.0 w/w% of GO, GF, or PG against Phi6 bacteriophage after 24 h. Error bars represent standard deviation (*n* = 3). *P* values of <0.05 compared to the control are indicated with an *.

As shown in Figure 9, pure PCL fibers (loaded with 0.0 w/w%) showed a slight reduction in virions (13.08 ± 1.38%). This is likely owed to the lack of host cells in the PBS to allow for viral survival and proliferation.

As seen in Figure 9, all nanocomposite fibers showed antiviral activity at the highest concentration tested. After 24 h of exposure, PCL fibers containing 4.0 w/w% of GO nanoparticles showed the strongest antiviral activity, with an average viral reduction of 33.08 ± 1.21% (*p*-value = 0.0008). PCL fibers loaded with GF and PG at the same concentration, showed similar but slightly lower antiviral activity, with reductions of 31.2 ± 1.11 and 30.5 ± 2.0%, respectively. Fibers containing the lowest nanoparticle concentration (0.1 w/w%) exhibited viral reductions of 13.4 ± 1.9, 15.4 ± 4.9, and 14.01 ± 1.8%, for GO, GF and PG, respectively (the difference between the pure fibers and loaded fibers is not statistically significant for all materials at this concentration). At a concentration of 0.5 w/w% of GF or PG, fibers showed a statistically significant viral reduction when compared to the control. Whereas GO fibers only showed a statistically significant reduction at a concentration of 1.0 w/w% or more. This indicates that porous-like structures are more effective at lower concentrations. Whereas at higher concentrations, solid structures are more effective, likely due to their increased exposed surface area.

Overall, the results shown in Figure 9 show the antiviral activity of all materials tested to be concentration-dependent. Increasing the loading increases the concentration of graphene-based nanomaterials on the fiber surface, therefore increasing the area of exposed material.

### Discussion

It has been explained in previous studies that graphene and its derivatives inhibit bacteria by many different mechanisms (57–60). The PG, GO and GF nanomaterials of the electrospun nanocomposite fibers included in this study were mostly embedded in the fibers. Therefore, reductions in direct action mechanisms may occur with nanomaterials. While this result may increase the biocompatibility of the nanofibers used, it may cause a decrease in antimicrobial activity. The oxidative stress mechanism is one of the basic mechanisms of graphene-based nanomaterials (57). In addition, it is stated as another mechanism that the graphene nanomaterials suspended on the fiber surfaces cause the loss of substances inside the cell by direct contact and the effect of the microbial membrane of their sharp edges (35). Finally, another mechanism called wrapping is the model in which nanomaterials in the environment encapsulate and isolate microbes (58). In this study, PG and GF nanocomposite fibers might inhibit viruses with their nanosized pores, wrapping and 3D sharp edges, while GO might increase antiviral activity mostly with the oxidative stress effect. However, the viral mechanisms of graphene-based materials still are not clear enough and should be investigated in more detail.

In antibacterial nanofiber studies, GO was mostly preferred because of its oxidative stress physicochemical properties and high biocompatibility. However, in general, GO polymer nanocomposites, which exhibit dose-dependent and time-dependent antibacterial activities (20), are more efficient at higher concentrations. GO toxicity has been found to be safe within a certain range in the human body (61). In this study, graphene-based materials at selected concentrations are limited to a maximum concentration of 4 w/w% in biomedical research to prevent harm to humans. Compared with the antiviral activity of GO nanocomposite fibers in previous studies, 4 w/w% GO was found to have a slightly higher antiviral effect in this study, 28.9 ± 1.2 and 33.08 ± 1.21%, respectively (18). The main reason for this can be explained as the fibers (0.53 ± 0.20 μm at 4 w/w% concentration) are thinner than in a previous study (1.55 ± 0.9 μm at 4 w/w% concentration) and therefore have a higher surface area to volume ratio (18). It is known that as graphene concentration of nanofibers increases, biocompatibility may decrease, and thus toxicity may increase.

The antiviral activity of GO has been investigated in previous studies (18), and during the COVID-19 pandemic graphene derivatives have been thought of as promising materials for the formation of antiviral fibrous mats. Even though this study was conducted against Phi6 bacteriophage, the data obtained indicate that graphene-based materials are potential antiviral candidates. Therefore, it has an important role for PPE used in preventing the spread of any viral infections.

Since PG, GO and GF nanomaterials are not completely soluble in acetone, they are dispersed and form a suspension. If the graphene derivatives in the formed polymer nanocomposite suspensions are not sufficiently dispersed, they may undergo agglomeration (39, 62). Agglomeration is seen in fibers with large bead formation. However, the reason for all of the beads formed may be not only due to the agglomeration of the particles but also to the electrospinning parameters. Another limitation is that nanomaterials are embedded in the fiber and not on the surface. Graphene-based material on the surface may show a higher antiviral effect, but for example, when used in face masks, it may also have a toxic effect as it can deposit in the lungs when inhaled directly. To prevent this, various surface modification methods such as electrospraying deposition can be tried.

Finally, electrospinning is a system that provides fiber formation from polymer solutions in the high electric field created by a high voltage power supply. At the same time, graphene and its derivatives have a high electroconductivity. Graphene-based materials in nanocomposite solution may be affected by the high voltage applied during nanofiber production and may not adhere to the fiber surface. In such cases, as the concentration may decrease, the antimicrobial activity will also decrease. At the same time, using the entire solution in the syringe in fiber formation is another important point. Since graphene is not completely dissolved in solution, it may collapse and remain in the syringe. This can likewise affect the amount of concentration. Therefore, it should be ensured that all the solution in the syringe and tube is used.

## Conclusion

In this work, morphology, chemical analysis and the virus inhibitory properties of 0.1, 0.5,1, 2, 4 w/w% concentration PG, GO and GF-loaded fibers were compared. SEM, FTIR and XRD characterizations were applied to the nanofibers. According to the results, the ultrafine fibers obtained mostly have porous surface properties and the mean diameter of all fibers was measured at around 460 nm. It has been observed mostly that the general trend is an increase in nanofiber diameters as the nanomaterial concentration increases. PG, GO and GF nanofibers showed antiviral activity against the SARS-CoV-2 surrogate and the highest inhibition was recorded as 33.08 ± 1.21% after 24 h. In this present study, PG and GF antiviral properties were investigated for the first time. The data showed that overall graphene-based nanomaterials are promising for biomedical applications such as PPE against the ongoing COVID-19 pandemic.

## Data availability statement

The original contributions presented in this study are included in the article/supplementary material, further inquiries can be directed to the corresponding author.

## Author contributions

SGE performed conceptualization, resources, visualization, analysis, and wrote the original draft. TAT provided resources and characterization, also reviewed the original draft. ME was involved in the conceptualization and overall supervision, reviewed and edited the manuscripts. RKM wrote the original draft and was involved in the conceptualization and overall supervision, reviewed, and edited the manuscripts. All authors contributed to the article and approved the submitted version.
